# Life and mental health in limbo of the Ukraine war: How can helpers assist civilians, asylum seekers and refugees affected by the war?

**DOI:** 10.3389/fpsyg.2023.1129299

**Published:** 2023-02-17

**Authors:** Gulnaz Anjum, Mudassar Aziz, Hadar Khasrow Hamid

**Affiliations:** ^1^Department of Psychology, University of Oslo, Oslo, Norway; ^2^Department of Psychology, Kwantlen Polytechnic University, Surrey, BC, Canada; ^3^Department of Psychology, Simon Fraser University, Burnaby, BC, Canada

**Keywords:** Ukraine war, war refugees and asylum seekers, mental health and psychosocial programs, psychotherapy, helping Ukrainians, life in limbo

## Abstract

The terror spread by the war disrupts lives and severs families, leaving individuals and communities devastated. People are left to fend for themselves on multiple levels, especially psychologically. It is well documented that war adversely affects non-combatant civilians, both physically and psychologically. However, how the war puts civilians’ lives in a limbo is an under-researched area. This paper focuses on three aspects: (1) how the mental health and well-being of Ukrainian civilians, asylum seekers, and refugees are affected by the war caused limbo; (2) what factors affect this process of being stuck in the limbo of war; and (3) how psychologists and helpers in the war-ridden and host countries can provide meaningful support. Based on the authors’ own practical work with Ukrainian civilians, refugees, and professional helpers during the war, this paper provides an overview of multi-level factors that impact human psyches in a war, and possible ways to help those who are living in the war limbo. In this research and experiential learning-based review, we offer some helpful strategies, action plans, and resources for the helpers including psychologists, counselors, volunteers, and relief workers. We emphasize that the effects of war are neither linear nor equal for all civilians and refugees. Some will recover and return to a routine life while others will experience panic attacks, trauma, depression, and even PTSD, which can also surface much later and can prolong over the years. Hence, we provide experience-based ways of dealing with short-term and prolonged trauma of living with war and post-traumatic stress disorder (PTSD). Mental health professionals and other helpers in Ukraine and in host countries can use these helping strategies and resources to provide effective support for Ukrainians and for war refugees in general.

## Introduction

The war in Ukraine started on 24 February 2022 and has since caused extensive human suffering and destruction. The war in Crimea and Eastern Ukraine goes back to 2014, and had already resulted in many deaths, large groups of internally displaced people, and psychosocial problems. The Russian invasion in 2022 has led to the world’s largest war led humanitarian and wmental health crises among Ukrainians. Presently, there has been extensive damage in many Ukrainian regions to people’s homes, public buildings, health facilities, and infrastructure. There are reports about atrocities committed by the Russian army on Ukrainian adults and children alike, including unprovoked killings of civilians, torture, and rape (United Nations High Commissioner for Refugees, 2022a). According to UNHCR’s report the number of displaced people around the world exceeded 84 million people in 2021 and 26.6 million of them are refugees and 4.4 million people are asylum-seekers (United Nations High Commissioner for Refugees, 2022a). These numbers were the highest that have been recorded in the last 20 years. The Ukraine war has produced even more alarming numbers and a concerning situation in this respect.

Ukrainian women, children, and elderly have been hiding in basements and underground shelters, with little access to water, food, and daylight. Families have been separated, and family members, friends, and neighbors have been killed or wounded. As of November 29th, 2022, more than 7.8 million people have fled the country (United Nations High Commissioner for Refugees, 2022b) and around 8 million people are internally displaced. Among Ukrainian refugees, many reside in nearby countries, and almost 1.6 million reside in Poland (United Nations High Commissioner for Refugees, 2022b).

The aims of this paper are three-pronged. First, review research regarding the factors that create a life in limbo experience for war victims. The paper highlights how war contributes to the refugees’ experience of the state of limbo. Second, we review multi-level factors that affect the mental health and well-being of refugees and asylum seekers in the context of the Ukraine war. Specifically, the risk and protective factors at physical, personal, social, and institutional levels are explored. We provide a model that considers multi-level factors that influence the mental health of refugee claimants and asylum seekers. Third, we provide experience-based suggestions and therapeutic approaches to help those working with war refugees.

## War trauma and refugee mental health: Life in limbo

There are many physical and psychological reactions to war trauma that are common human responses to extreme and cumulative stress. Nevertheless, they are highly unpleasant and often a cause of concern to the person experiencing them, and to their family. It may help to know that these reactions are natural responses to having experienced severe threat. It is also the case that the trauma of war and related stressors do not cease once people leave the war situation because stressors of finding a new host country and home can exacerbate some of these concerns.

### War trauma and refugee mental health

Civilians from war zones experience multiple traumas and adversities that may contribute to the psychological reactions or health problems that are found in refugee populations. These include adverse and traumatic events during episodes of bombing, highly threatening escape during war or conflict, physical and sexual violence related experiences, human rights violations, and dangerous situations during heavy fighting. In our recent interactions with Ukrainian war-affected persons, we were warned of high levels of psychosocial stress in the place where they found refuge, whether in internally displaced persons’ shelters within their country, or as refugees in a foreign country.

People react differently in the face of traumatic situations such as war (Opaas and Hartmann, 2021). They freeze, they cannot move or act, they fight the situation, or they flee. These are instinctive reactions meant for survival, depending on the circumstances. In the aftermath of dangers such as war, people also react differently depending upon their personal characteristics and of the stressful events. Some of the personal factors that may increase the risk of stronger or more prolonged psychological or psychosocial difficulties after trauma of war include being a female, being a child and of older age, poverty and socioeconomic difficulties, previous or current psychological problems, family dysfunction, previous trauma exposure, and genetic predisposition to stress and depression (Opaas, 2022).

Characteristics of the stressful events experienced during the war are related to stronger and more prolonged difficulties such as: intentional acts of violence rather than accidental, life threat, the extent of exposure to combat and injuries in war, witnessing death, loss of a loved one in war, life-threatening situations during bombing, lack of control with uncertainty and unpredictability, and long duration or greater frequency of traumatic events during the war (Opaas, 2022). These adversities accumulate and war-led trauma makes people more vulnerable to developing mental health and psychosocial problems. If one already suffers from a mental health disorder, being exposed to war trauma and adversities might make it even worse (Bhui, 2022). The next threatening experience may just be one too much for the person (Briere and Scott, 2014).

Clinical researchers have indicated a high prevalence of mental health challenges in refugee populations. For instance, Steel et al. (2009) report that 30% of refugee adults experience post-traumatic stress disorder, and more than 30% experience major depression. In addition to the pre- and post-resettlement stressors, which are rightly considered key factors in the refugee population’s mental health outcome, other factors that are specific to the process of being a refugee, the asylum-seeking process, and the transition period from trauma to settlement are also major influencers (Khawaja et al., 2008). The asylum and refugee status-seeking process is categorized as “living in a state of limbo and uncertainty” (Posselt et al., 2018) which takes its own toll on mental health. Due to the large-scale refugee population who spend time in life-threatening war situations and waiting, it is imperative to underscore the effect of the limbo state on mental health.

Previous research has shown that historically new waves of refugees from conflict zones have evoked opposition and resistance in host nations and host countries (Arakelyan and Ager, 2020). Stressors related to forced displacement such as separation from family and one’s own culture are exacerbated by the new conditions and different culture in the host country. Additionally, the personal and individual experiences add to the complexity of the situation and the overall mental health outcomes of the refugee and asylum seeker population. Thus, refugees and asylum seekers find themselves between the *mortar* and the *pestle* with limited choices while experiencing war and going through asylum processes in transitional or host countries which results in uncertainty and waiting.

The mental state of war victims and refugees is characterized by life with precarious situations, seeking status, and sometimes without any certainty in formal immigration status in the host country. For the purpose of this paper, precarious status refers to the situation when an individual has to decide between living in a risky (war) environment or dealing with the risk of leaving everything they have ever known behind them. In other cases, precarious conditions can also be the situation where an application for refugee status has been submitted which is still in process, or the application is denied. Thus, the individual is not officially accepted as a refugee in the host country. Both cases create a situation with life in a war limbo.

### Living in a state of limbo: Bearing war and becoming refugees

Before we describe the state of limbo, it is important to differentiate between asylum seekers and refugee status of persons who flee conflict and war zones. An asylum seeker is an individual seeking international protection in countries with individualized procedures (Amnesty International, 2022). An asylum seeker is someone whose claim to become a refugee has not yet been decided by the country in which they have submitted their application. Therefore, every refugee is initially an asylum seeker but not every asylum seeker will ultimately be recognized as a refugee and receive legal protection and material assistance. An asylum seeker must demonstrate that his or her fear of persecution or risk to his life in their home country is well-founded.

According to Habitat for Humanity (2022), Refugees are people fleeing armed conflicts or persecution. Their situation is so perilous that they cross national borders to seek safety in other countries and become recognized as refugees with access to assistance from the host states and aid organizations. Being recognized as a refugee is determined through a legal process of Refugee Status Determination (RSD), which is used by the governments or UNHCR to determine whether a person seeking international protection is considered a refugee under national and international law. All refugees are primarily asylum seekers until they are recognized as refugees.

The state of asylum seekers in transition is described as a “state of limbo” (Solberg et al., 2021). At this stage, they lack formal status while awaiting their refugee claim to be finalized. Haas (2017) describes this stage as “citizens-in-waiting and deportees-in-waiting.” Asylum seekers describe this stage as life being on hold and waiting for it to resume (Bjertrup et al., 2018). This places a great toll on psychological well-being. In addition to the uncertainty, this stage causes the feeling of regret and guilt due to separation from family, inability to care for family’s needs, and children missing out on their childhood and their future (Hoffman, 2011). Furthermore, the lack of control over their circumstances has a great impact on asylum seekers’ psychological well-being as life in this state has no direction (Haas, 2017). In other words, the process which is meant to be a temporary state causes trauma that could have a long-term effect on the individual and possibly even after the asylum is granted (also see Uphoff et al., 2020).

**Figure 1:**
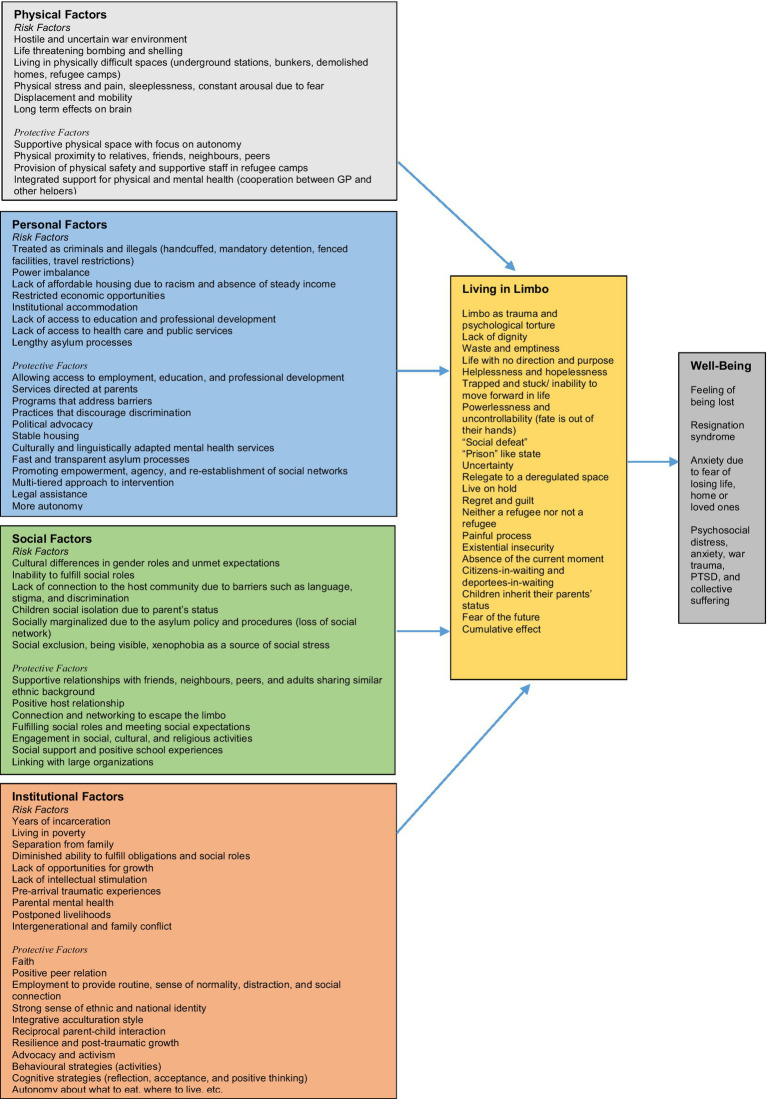
Multilevel factor affecting life and wellbeing of war civilians, asylum seekers, and refugees.

In war and other mass conflicts, or individually committed violence, the fear and suffering is caused either directly or indirectly by other human beings’ acts and decisions. Usually, the acts are intentional and willed. Examples of intentional acts of violence are armed attacks, torture, rape and other sexual assaults, domestic violence, and child abuse. Research has shown that violence caused intentionally by other human beings brings about the most severe reactions in those affected (Briere and Scott, 2014). The process and the effect of this limbo of being in the war is influenced by multi-pronged factors that are both outcome as well as cause of negative effect on mental health issues for refugees. These factors include physical, personal, social, and institutional factors. The summary of these multilevel factors is presented in Figure 1.

#### Physical factors

Being in a physically hostile war environment can be stressful and traumatic. Prolonged physical distress caused by sirens and bombing leads to distress and even trauma negatively affecting physical health and well-being. After experiencing severely threatening situations, some immediate physical reactions may be sleeplessness, nightmares, somatization (Balaban et al., 2005) restlessness, jumpiness, physical arousal, high bodily activation, being alarmed and on guard, feeling weak, and physical experience of numbness (World Health Organization, 2015). For children, physical threats lead to sleep disturbances, and bed wetting is also common (Almoshmosh, 2016). Severe or long-lasting stress can also cause physical diseases.

Compared to war-exposed civilians, refugees and internally displaced individuals’ experiences may be more traumatic. It is not only because of the situations that led to their displacement and exile but also because of physical stressors experienced in migrant and refugee camps, and during the process of resettlement. They exhibit high rates of stress, depression and PTSD, and other physical and psychiatric problems. This is particularly the case if they were physically tortured (de Jong et al., 2000). In a survey of Bosnians who lived in a refugee camp in Croatia, one-third of those who experienced more than six traumatic events were diagnosed with depression, and one-quarter of them had PTSD. More than 20 % of these refugees met the criteria for both depression and PTSD. Moreover, refugees with both depression and PTSD were five times more likely to report being physically disabled than refugees with no symptoms of psychological disorders (Mollica et al., 1999).

A study conducted by Gerritsen et al. (2006) indicates a higher prevalence rate of poor physical health conditions among Afghan, Iranian, and Somali asylum seekers in the Netherlands compared to refugees. Participants in this study reported suffering from chronic neck and shoulder pain, back pain, and headaches. Along the same lines, Laban et al. (2008) findings suggest positive correlations between the length of the asylum-seeking process and disability as well as chronic physical complaints among Iraqi asylum seekers in the Netherlands. Additionally, the longer the asylum-seeking process was, the lower the quality of life of the participant was.

A study conducted by Fnaskova et al. (2021) with Holocaust survivors showed lifelong changes in the brain. Holocaust survivors experienced significantly higher levels and frequency of depression symptoms, posttraumatic stress symptoms, and lower levels of well-being. Their MRIs showed a lifelong neurobiological effect of extreme stress of the war and trauma of the Holocaust. There was significantly reduced gray matter corresponding to the map of the stressed brain structure. Despite showing good adjustment to post-war life conditions, the reduction of gray matter was significantly expressed. More importantly, these patterns were present in the subgroup of participants who survived the Holocaust during their childhood suggesting that these are lifelong psychological and neurobiological changes. War and survival related extreme stress has an irreversible lifelong impact on the physical composition of gray matter in the brain of war survivors (Fnaskova et al., 2021).

Many civilians after war and asylum seekers express their complaints as physical problems which in many cases cannot be physically diagnosed because these complaints are sometimes associated with heightened stress (Hondius et al., 2000). In such cases, the general practitioner should be consulted and informed by mental health professionals emphasizing how psychological problems are deeply linked with physiological problems (Laban et al., 2007). In many refugee camps and healthcare workers warn about the effects on mental health due to a strict reception policy which should be reported to the policymakers (Reijneveld et al., 2005).

Broken homes disrupted infrastructures and resulting displacement can also lead to loss of sense of place, and disrupted identity among people who experience war-related disasters. Many war victims, asylum seekers, and refugees will experience cognitive consequences as a result of war-related physical factors. These consequences include problems concentrating, problems remembering things in daily life, problems learning new things, loss of creativity, or loss of interest in activities that were previously joyful. Some will feel rejected, forgotten and let down by God, or may lose faith, temporarily or in the long term. Others will turn to God and feel strengthened.

Reactions to trauma are often presented as emotional, physical, or cognitive. It is often difficult to distinguish between them. We are one organism, and what is happening to us affects our whole body and mind, our feelings, our ability to think, concentrate and learn, our spiritual self, and our relations to others. Emotional reactions include: shock, disbelief, grief, anger, irritability, anxiety, fear, detachment, and insecurity. Women may be more likely to have internalizing symptoms (Hodes, 2022). Already existing mental health and social issues might amplify when exposed to trauma as well (World Health Organization, 2015).

#### Personal factors

Due to individual differences and unique circumstances during the process of war and displacement, refugee individuals who have not received legal status in a potential host country have a variety of personal risk and protective factors. These factors influence how the transition state is experienced. The individual risk factors are associated with feelings of uncertainty and insecurity due to unresolved legal status. As a result, the asylum seekers live in fear, isolation, and poverty as they spend years of incarceration and are separated from their families (Hoffman, 2011). Additionally, the asylum seekers report feeling of powerlessness and lack of control over their situation as they wait for decisions to be made about their future (Hoffman, 2011; Bjertrup et al., 2018).

Other key factors that contribute to psychological deterioration in asylum seekers and refugees include lack of procedural knowledge, cultural differences, intellectual stimulation, and opportunities for growth (Simich et al., 2010; Bjertrup et al., 2018; Byrow et al., 2020; Pluck et al., 2022). This is due to a lack of access to education and employment. Parents report feeling guilty as their children miss out on their education (Hoffman, 2011) and incompetent as they become unable to fulfill family obligations (Simich et al., 2010). Lack of access to employment has not only economic consequences but also psychological consequences as it is a source of routine, stability, connection, and social network which contributes to feeling safe and secure (Hoffman, 2011). Thus, the process of waiting adds to the psychological deterioration of asylum seekers as it creates a state of uncertainty, insecurity, isolation, and hopelessness. This diminishes the livelihood of individuals as they feel that their lives are on hold.

There are also protective factors that can aid the mental well-being of asylum seekers by increasing resilience when facing uncertainty and hopelessness. For example, asylum seekers try to bank on their faith, spirituality, and religious practices (Kramer and Bala, 2004; Posselt et al., 2018). This provides a sense of normalcy as well as opportunities for social connections. Furthermore, asylum seekers also adopt behavioral strategies such as physical activities, engaging in hobbies, and watching movies as a source of distraction from their current state. Also, they report adopting cognitive strategies such as acceptance, positive thinking, and finding meaning in suffering to normalize and minimize the severity of their situation (Posselt et al., 2018). Thus, while going through the lengthy asylum process, refugee claimants are actively trying to resort to strategies and practices that tap into their mental well-being.

#### Social factors

There are risk and protective factors at the social level that also influence the mental health of asylum seekers. One of the key social risk factors is the isolation and lack of connection to the host community due to barriers such as stigma, competency in the host country’s language, and discrimination (Bernhard et al., 2021; Solberg et al., 2021). Additionally, the host community might be unwelcoming due to the surge in refugee population numbers which could lead to further exclusion and marginalization of the refugee population. Other risk factors that contribute to deteriorated psychological well-being during the asylum-seeking process include the inability to fulfill social roles and social expectations (Simich et al., 2010). Asylum-seeking children also report being bullied, excluded, and marginalized due to their parents’ status (Bernhard et al., 2021). Thus, the asylum-seeking process and the uncertainty of living in a state of limbo hinder social integration in the host community.

It is critical to note that the trauma of war can lead to a wide range of emotional and psychological reactions. Among Ukrainian refugees who are living in this limbo, we see higher levels of helplessness, feelings of hopelessness, anxiety, and fear, especially women with children. Additionally, women may also face additional challenges such as sexual violence, exploitation, and discrimination, in the context of the ongoing war in Ukraine.

The ongoing war in Ukraine has had a significant impact on the mental health and well-being of women living in affected areas. Similar to studies done in the previous war contexts, research conducted by Fel et al. (2022) in the war-torn Ukraine has shown that women are at a higher risk of experiencing post-traumatic stress disorder (PTSD), and other mental health issues. This research indicates that while predictors of posttraumatic stress can be multifold including are loss of a loved one and place of residence, the presence of material security lowers PTSD, but only among those who have no children. In the case of Ukrainian women who are living in limbo of the war, higher PTSD is the result of not only the violence and damage caused by war but also of other stressful circumstances associated with the social and financial conditions of living in a war.

These findings of more disproportionate effects on women bearing the brunt of war related anxiety, stress and trauma are also supported by a UNPF report (United Nations Population Fund, 2018). This report found that the women reported higher levels of physical and emotional abuse, as well as negative effects of displacement and loss of social support. Our own recent work with Ukrainian refugees in Norway, Canada, and Poland shows that these negative consequences persist in refugee Ukrainians who have fled their homes and who now live in other countries. Furthermore, discrimination and gender-based violence (GBV) are also reported to exacerbate in the context of war. In research led by Capasso et al. (2022), patterns of GBV were explored in the high conflict-zones of Ukraine. In an in-depth analysis of the internally displaced women who were receiving psychosocial support services, it was found that women living in conflict-affected areas in eastern Ukraine reported high levels of gender-based violence, displaced women were suffering from GBV nearly three times more than non-displaced residents. This study underscores that nearly half of the displaced women experienced intimate partner violence and psychological abuse. Displaced women living inside Ukraine were more likely to report non-domestic GBV incidents involving sexual violence and were highly vulnerable compared to non-displaced women. It is important to note that sexual violence, exploitation, and discrimination are underreported in conflict-affected areas and that the actual numbers may be higher. It is also important to have specialized services and support for women who have been affected by gender-based violence, and to raise awareness about the issue.

Another study by Roberts et al. (2017), collected evidence on the mental health and psychosocial support needs of internally displaced persons (IDPs) in Ukraine. The purpose of the study was to help inform relevant policies and programs with data on the burden of mental disorders, and to design appropriate trauma-informed mental health and psychosocial support responses for millions of IDPs inside Ukraine. This showed 32% prevalence of PTSD, 22% prevalence of depression, and 17% prevalence of anxiety. All of these psychological stressors were significantly higher among women than in men. These findings recommend that in addition to refugees and asylum seekers, IDPs must be considered as one of the highly vulnerable groups for mental healthcare, and social support provision by the relevant agencies in Ukraine. These additional layers of vulnerabilities can further compound mental health issues for refugee women. Therefore, it is vital to have increased access to mental health services and support for women in host countries, as well as awareness-raising and education about the mental health consequences of the Ukraine war and the displacement of Ukrainians.

In addition to the risk factors, there are some protective factors at the social level that support asylum seekers to cope with the uncertainty that is created by their lack of legal status. Friendships with peers and adults sharing similar ethnic backgrounds and positive host relationships aids in better mental health of refugee seekers (Arakelyan and Ager, 2020). Social connections and engagement in social activities serve as an outlet for distraction and sharing of experiences as well as emotional and practical support (Posselt et al., 2018). Additionally, the re-establishment of social networks also improves refugee children’s school experiences (Measham et al., 2014). Thus, the host communities’ support and willingness to include refugee individuals improve refugees’ psychological outcomes during the period of uncertainty and hopelessness.

Depending on age, personality, and the context, some individuals become more dependent and clinging, some become easily irritated or angry, some become very quiet and keep difficult experiences and feelings to themselves to avoid burdening loved ones, and some isolate themselves to avoid trauma reminders or feelings of social shortcomings. Some cannot bear to be physically intimate after severe experiences, others seek relief through intimacy. Some do a mix of all of these. After traumatic experiences, family members may become closer to one another or may drift apart.

#### Institutional factors

Many factors at the institutional level contribute to the refugee population’s experience of the state of limbo that exacerbates their stress and war trauma. Institutional factors such as policies and procedures around the asylum processes place refugees in extremely difficult situations, for instance, they are put into refugee camps with restricted mobility (Pluck et al., 2022). Asylum seekers are publicly treated with many forced institutional practices, especially perceived as societal threats. Other institutional policies include restricting asylum seekers’ access to health care, employment, education, and social services such as subsidized child-care (Bjertrup et al., 2018). These institutional restrictions in the host countries create barriers to affordable and stable housing causing more strain on the physical and mental health of refugees. Furthermore, the process takes years and lacks transparency which further affects mental health (Measham et al., 2014). Hence, the asylum-seeking process creates additional stressors that exacerbate stressors related to integration which impact refugee populations’ well-being.

Policies and interventions can utilize institutional protective factors that tap into better mental health of asylum seeker- and refugee populations. These protective factors include fast and transparent asylum processes, practices that promote empowerment, agency, and re-establishment of social networks, and legal assistance (Bjertrup et al., 2018). Refugee populations also benefit from more autonomy in decision-making related to where to move and live, what to buy, and what to eat. Another important institutional-level protective factor is access to employment, training, education, and political advocacy (Solberg et al., 2021). Utilizing protective institutional factors will preserve the lives of asylum seekers and refugees from being stuck in the uncertainty of limbo, and they have the potential to minimize the negative impacts of the war-related trauma and safeguard their well-being.

### How to be helpful: Practical guide for working with war civilians and refugees

**Figure 2:**
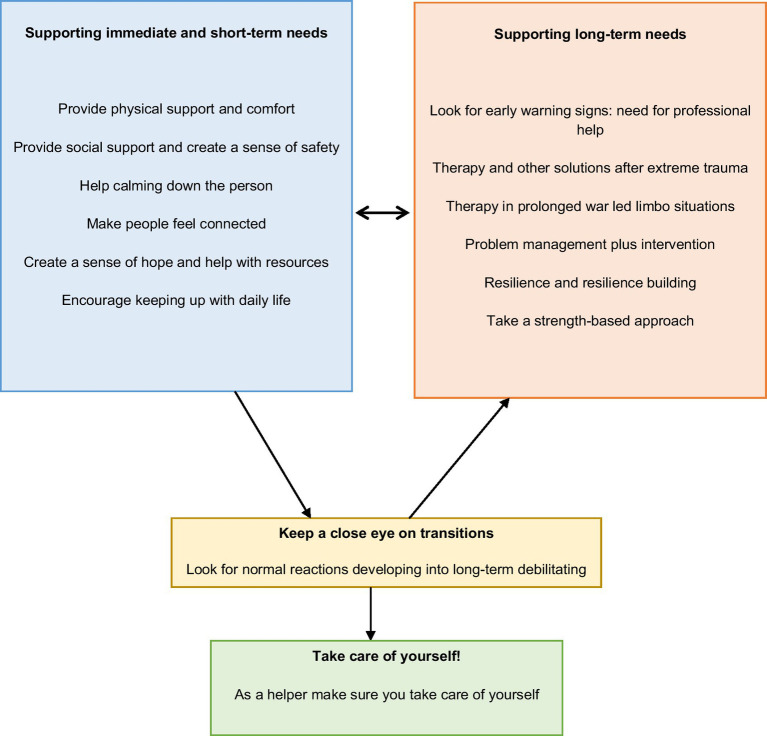
How to be helpful: Practical guide for working with civilians, asylum seekers and refugees of war zones.

During our work with Ukrainian civilians in Ukraine and war refugees in various host countries in the EU between May–October 2022, as psychologists, we worked with several trauma therapists, counselors, and helpers in Norway. Based on our reflections about how we could be most helpful, and after discussions with Ukrainian psychologists and psychology students, we were able to prepare a set of effective actions that could count towards providing help and protecting people from war trauma (or at least avert its exacerbation). After prolonged interactions and conversations with several trauma researchers which spanned over 6 months, we concur that there is some practical advice that can be provided to an individual who seems to become anxious and overwhelmed after war trauma. This is also what helpers can do for themselves when they feel overwhelmed. If you are a therapist, a trauma psychologist, a counselor in a war zone or a refugee camp, or another helper who is offering short-time support to people who have suffered war-related traumatic events, here are some of the very actions that can be helpful. In order to provide the needed support, helpers need to differentiate between immediate and long-term support and be open to using different strategies depending upon persons’ needs. See Figure 2 for a summary of the short-term and long-term needs of civilians and asylum seekers. It is important to note that these needs may transition from short-term to long-term needs.

### Supporting immediate and short-term needs

In most people, the reactions to trauma may be strong in the beginning, and then they slowly diminish. In most cases, low-threshold psychosocial support is sufficient.

#### Provide physical support and comfort

Offering a comfortable sitting space, something to drink, and maybe a blanket might seem like small acts but according to our experts, they matter a lot because being in a culturally alien place is a potential cause for insecurity and mistrust (Gartley and Due, 2017). Introduce yourself, your relevance with your role as a helper, and what you know about the person’s situation. Tune into the person with the tone of your voice, your attention, facial expression, and bodily posture. Ask fewer questions and focus on listening. Be there physically and mentally and listen to them with full attention, and give empathic feedback (Sripada et al., 2011). Acknowledgments such as “It must have been very scary,” “That must be so hard,” and “I am so sorry for your loss” could be very helpful. Be reassuring and honest: “It’s OK, now you are here,” “You are safe now.” Do not worry if the person starts to cry or you cry (Blume-Marcovici et al., 2013; Capps et al., 2015). You are there to support and the person is counting on your support.

Sometimes, for persons with repeated traumatic events, i.e., repeated shelling in their cities, and who feel extremely unsafe, physical exercises can help (Dhillon et al., 2019). For instance, we can ask these persons to stand on their feet or sit straight with their feet firmly on the ground. Look at objects around them, or people, anything that looks normal. Breathe deeply and slowly because that can be helpful in reducing stress (Çesko, 2020). Then ask them to move their limbs, shoulders, and body. Follow this by asking them to stretch their arms up and out to the sides, and move around if they have energy. Sometimes it can help to be with someone they know, and they can trust. Physical movement, especially walks, can help people in taking their minds off trauma, and it has been found to be helpful among refugees (Zschucke et al., 2012; Sjögren Forss et al., 2021). Physically writing down recurring memories, thoughts, and dreams that bother them can be helpful for them. Lastly, trying to resume their ordinary activities, if possible, can be helpful.

#### Provide social support and create a sense of safety

During a crisis or a war, many people experience a total lack of safety. It is therefore important to restore some sort of safety as much as possible (Hobfoll et al., 2007). Bringing someone away from an acute situation (a war front, a traumatizing scene, etc.) can be helpful (Solomon and Benbenishty, 1986; Solomon et al., 2005). It can also help to provide a voice within the immediate environment that can restrict the narrative within limits. This is to increase the sense of safety and reduce the fear of the threat still being around. However, it is important not to alter the information just to create a sense of safety, as it might backfire. It may be wise to encourage the survivors to stay away from reading and watching the news, particularly children, as media can potentially increase the sense of threat if reported a lot (Hobfoll et al., 2007). If a person seems to feel anxious and depressed when talking about the trauma, do not pressure them to talk about it. In addition to this, it can be helpful to disrupt harmless pictures or items which have through the trauma been linked to danger or stress (Hobfoll et al., 2007). Try to link those pictures/items back with something harmless or good. While doing this, it is important to create a sense of trust in you as an organization in order to further engage in successful interventions (Winer, 2021).

As a helper one can get emotional and it is OK if you get some emotional reactions yourself, like tears in your eyes. It is ok if the person you are helping, sees that what they have suffered makes you feel angry or sad. This honest expression can create a safe space for the refugee. However – stay calm – calmer than the person you want to help. Give relevant information about normal reactions, how to handle stress, and where practical help may be obtained. In the moments you feel helpless, do accept your own feelings of helplessness. Even though you may feel you have nothing to offer, what you do is helpful. After helping others, if possible, do something good for yourself.

#### Help calming down the person

There are a variety of ways to help people calm down. This is to reduce arousal levels, which at a high level might contribute to the onset of anxiety disorders down the road (Shalev et al., 1998; Bryant et al., 2003; Hobfoll et al., 2007). First and foremost, help people with immediately needed resources. This helps relieve some of the immediate concerns one faces during a crisis (Hobfoll et al., 2007). To help the individual or group to calm themselves down, one can encourage steady breathing. Breathing is a simple technique that is used to get individuals to breathe deeply and avoid hyperventilating or dissociating (Foa and Rothbaum, 1998; World Health Organization, 2016). Reassuring people’s stressors and feelings, and letting them know their reactions are normal can also help calming (World Health Organization, 2015). One can also try to involve the individual or group in an uplifting activity, to distract and move the focus. By engaging in an activity where one learns a new skill, one also increases the sense of self-efficacy. This can increase a sense of control and provide opportunities for small wins (Baum et al., 1993).

#### Make people feel connected

Connecting with others is important to restore normal interaction and well-being (Almoshmosh, 2016; World Health Organization, 2016). This can also help prevent future mental health issues (Hodes and Hussain, 2020). Help the survivors to connect and link with family, friends, and other loved ones. Increase the frequency and quality of their connection, by facilitating to the best of your ability (Hobfoll et al., 2007; World Health Organization, 2016; Hodes and Hussain, 2020). Where family reunion is not possible, other care arrangements should be in the best interest of the person and it should be a priority to provide the option of returning to immediate or extended family (World Health Organization, 2015). If possible, create communal spaces such as playgrounds, church, entertainment places, sports fields, etc.

In addition to connecting with others, mentoring services and community solidarity activities can be helpful areas to engage civilians, asylum seekers, and refugees. Also, creating a welcoming and supportive environment around the survivors in the local communities is important to increase the sense of belonging and making connections (Almoshmosh, 2016; Hodes and Hussain, 2020; Bhui, 2022). This is particularly helpful if one is in a host country or a different community than usual (Almoshmosh, 2016). When designing interventions, be conscious of negative social influences such as in-group/out-group issues, mistrust, and impatience with recovery (Hobfoll et al., 2007). In addition, partnering with community members who are bilingual and/or bicultural speaking Russian or Ukrainian can foster connectedness and more trust while engaging in interventions.

#### Create a sense of hope and help with resources

By providing hope, one can increase self-efficacy, and well-being (Snyder, 2002). Hopeful people believe that difficult situations can change for the better whereas those with strong efficacy believe they possess specific skills to make changes in those situations. Therefore, in bad situations, individuals with a sense of high self-efficacy and hope show better determination in seeking their goals (Rand, 2018). Research in positive psychology has demonstrated that hope is a protective factor that may positively impact a person’s psychosocial and spiritual development, including their perceived level of self-efficacy, effective coping, resilience, and personal growth (Snyder, 2002). It can be helpful to provide services that help them build hope and efficacy for getting their life back on track such as housing, schooling, legal support, employment, help with insurance reimbursement, or other financial obstacles (Hobfoll et al., 2007; World Health Organization, 2015; Hodes and Hussain, 2020). This way, the road to normal life is shorter and progress is seen by the individual/group. Make sure the information is conveyed and understood by everyone, including illiterates, people with disabilities, and children (World Health Organization, 2015).

In addition, the surrounding community like media, universities, churches, schools, managers at workspaces, and other natural community leaders should be able to help with linking resources, setting positive goals, and making meaning to their everyday life. Particularly for children, teachers play an important role in helping identify needs and guiding the children and parents to the appropriate resources (Hodes, 2022). School-based support can also be helpful for children, particularly when learning a new language and adapting to a new culture (Winer, 2021; Hodes, 2022). One can also help the individual identify and define a current problem themselves, and brainstorm together to find a solution. A solution to the entire problem might not occur right away, so it is important to validate and focus on what can be done in order to avoid helplessness (World Health Organization, 2016). Make sure the person also has some sort of autonomy to create a sense of personal control.

#### Encourage keeping up with daily life

If an individual is lacking motivation or seems to withdraw from daily actions (showering, hanging out with friends, going outside) as a consequence of the trauma, it is important to address this (World Health Organization, 2016). It can be helpful to address this problem with the person and help them understand that “do first and the motivation or positive feelings will follow,” rather than waiting to engage in the activity until they feel motivated. An action plan with small steps can be helpful to slowly get the person back on track (World Health Organization, 2016). Following up on the plan to address progress might be helpful as well.

When helping refugees with resources, interventions and potentially diagnosing, it is important to put our own biases aside and try to understand where they are coming from (Winer, 2021). Symptom expression varies between cultures (Bhui, 2022), so it can be helpful to get to know the individual by having a well-structured interview before jumping to interventions. If you are trained in conducting The Cultural Formulation Interview recommended by the DSM-5, it can be very helpful if providing therapy (Lewis-Fernández et al., 2020). The most important aspects are ensuring the patient’s specific story and culture is embraced, heard, respected, noted, and built upon (World Health Organization, 2015; Bhui, 2022).

### Look for early warning signs: Need for professional and log-term help

In some cases, people coming from war zones may show some warning signals that indicate the need for professional assessment and treatment. The following signs are important to observe extremely hard time sleeping; panic attacks; signs of apathy, longer stays in the bed, resisting food and drink, does not respond to anyone who is trying to make contact; suicidal ideation or attempts; rage towards others and threatening behavior, violent acting out or being a victim to violence in the present; extreme confusion, possibly dementia; pointless and hectic behavior; psychotic symptoms (delusions and hallucinations).

In some cases, people also experience personality changes after adverse events. For instance, post-war people’s whole personality changes from how they used to be before the traumatic events: The person may develop chronic changes that can affect their personality including but not limited to developing a chronic feeling of being on guard of imminent and lurking threats; development of mistrust towards other people; social withdrawal; feelings of emptiness and hopelessness; and feelings of being alienated from other people and the world around. Such personality changes may pass within some weeks, or a few months. However, sometimes these changes take hold. Major happy life events and a loving and safe relationship may counteract such distrust, alienation, and feelings of threat. But sometimes, psychotherapy over time is the only remedy.

#### Look for normal reactions developing into long-term debilitating states

Exposure to severe trauma may not necessarily lead to symptoms of posttraumatic stress disorder (PTSD). When people experience excessive stress, they react in different ways. Other than PTSD, people may develop: Depression, Anxiety, Complex posttraumatic stress disorder (CPTSD), Somatic complaints, Pain problems or physical disorders, Substance abuse, Personality changes, Dissociative disorders, or Psychoses. More often than not, people develop problems in more than one area, such as comorbid PTSD, depression, and somatic problems.

#### Therapy and other solutions after extreme trauma

Those of us who are in the position to provide psychotherapy over some time to someone affected by traumatic experiences, here are some suggestions from professional trauma therapists about providing support. More specifically, we recommend these actions that can be helpful. First of all, it is important to listen with kindness and be accepting by sharing empathic reactions to what the patient tells you. In a support role, be accepting of crying and other strong reactions, and stay calm and reassuring. The second mechanism of support is to orient reactions and feedback to here-and-now worries. For Instance, attending to the bodily stressors, including physical pain, sleeplessness and nightmares, and any need for medical checks because psychological stressors start expressing as somatic signs. Third, there is a need to make assessments and tend to psychosocial problems, depression, anxiety, or posttraumatic stress symptoms, and the most pressing worries, including for their children and loved ones. While working therapeutically with trauma, choose incidents that invade thoughts or appear in nightmares, and that presently bother the person the most. Especially for mothers with young children, the therapeutic approach can have beneficial effects for the adult refugees, and it could mitigate the impact of war related-trauma on women and their children (Bürgin et al., 2022).

To address trauma, you may use whatever method you are comfortable with or that you feel works with the particular patient: EMDR (eye movement desensitization and reprocessing), TF-CBT (trauma-focused cognitive-behavioral therapy), TST-R (Trauma System Therapy- Refugees), Problem Management Plus intervention (World Health Organization, 2016), Narrative Exposure Therapy (Robjant and Fazel, 2010) or just talk therapeutically with the person about the experience, gradually in more detail. For a start, nightmares may be somewhat easier to talk about and work therapeutically with than what really happened. Most of the time war refugees can get into therapy with multiple incidents. It is alright to start working with one incident at a time. There are also accumulative and sustained effects of working with one traumatic incident that may also be helpful for other incidents that are not dealt with in therapy.

Furthermore, as a therapist, writing down what they tell you, and then reading it out loud to them later in the session or at the next meeting, can be a strong emotional and painful experience, but also a deeply confirming and healing experience. Trauma may also disturb an individual’s perception and interpretation of events in daily life. If this seems to be the case, support the person’s reality testing and understanding of encounters with other people, events, and aspects of the society. This can be done by discussing current and troublesome experiences and relationships. You can also encourage patients to express their beliefs and impressions of the therapist. Confirm if their perception of the therapist is correct. Keep an open mind about experiences that cannot be easily confirmed or disconfirmed but discuss different alternative understandings. Last but not the least, in order to help, instilling hope can be achieved by mapping the individual’s personal strengths and skills.

#### Therapy in prolonged war led limbo situations

In a prolonged longitudinal study of psychotherapy patients with refugee and trauma backgrounds, Opaas and Hartmann (2021) found that many patients had suffered war-related trauma, persecution, torture, and sexual assault. The amount of war-related trauma that individuals had suffered was significantly related to posttraumatic stress symptoms, especially re-experiencing symptoms. Patients with childhood trauma in addition to war-related trauma, had more symptoms of mental health disorders and lower quality of life at the start of treatment than those without childhood trauma.

Opaas and Hartmann (2021) followed up on the former patients after three to 10 years. At group level, they found that the patients improved significantly over the first years. However, men did not respond to psychotherapy as much as women, and those with a higher number of traumas, those with experiences of torture, and those with childhood experiences of violence within the family had a harder time recovering from their mental health disorders than those with less trauma. This study is special because there are not many studies reported with such long follow-ups.

Trauma-focused personality assessment shows that the patients are characterized by varying levels of, or oscillating between, flooding and constriction, and by varying levels of trauma-related problems with reality testing. Because of traumatic experiences in the past, some may misread events, misinterpret situations, and other people’s motives and acts in the present. Opaas (2022) suggested that those whose reality testing and judgment of the present functioned well despite traumatic reminders, can improve more rapidly in therapy. Those who have more problems with perceiving objective reality may need a longer therapy, or therapy focused on discussing present relationships and experiences.

#### Problem management plus intervention

The problem management plus (PM+) is an intervention to provide individual psychological help for adults impaired by distress and trauma in communities exposed to adversity (World Health Organization, 2016). It can be provided by non-clinical professionals, which makes it convenient and accessible. The intervention consists of six modules to help an individual with problem management. These modules include managing stress, managing problems, getting going, keeping doing, strengthening social support, and staying well. However, it is important to note that we should not provide psychotherapy if the person is not in a stable situation. If the person is soon to be moved to a different facility or area where psychotherapy cannot be followed up, certain therapies like trauma-focused single-session interventions can do more harm than good (World Health Organization, 2015). Therefore, as helpers, we need to make sure when starting psychotherapy that follow-up sessions are possible to maintain.

#### Resilience and resilience building

Many people handle war trauma and other adverse life events with resilience, and without developing reactions that impede their everyday functioning (Bonanno, 2004). Some are able to handle repeated experiences of adverse life events without getting traumatized or without needing clinical help. Some, however, get strong reactions for a while, but then recuperate fully. Yet others will need short-term or longterm clinical and social support to build resilience after living through a war.

*Resilience refers to th*e individual’s ability to use resources within themselves and in the environment to overcome distress. Even if the individual does not fully have these capacities, resilience can be stimulated by active outreach and support from others. Relationships are important in times of extreme stress, and a single friendly person can make a difference. Research by Fernando (2006) found that among war-affected children in Sri Lanka, the children with higher resilience were better able to make sense of and accept their trauma of the war they had survived. Building resilience can transform negative thoughts and emotions into a more positive outlook. These findings were also supported by another study by Shoshani and Slone (2016) where they found adolescents’ resilience as a function of character strengths in the face of war and protracted conflict. War-related violent exposure was associated with psychiatric symptoms whereas resilience function of character was negatively associated with psychiatric symptoms. Thereby building resilience can be a potent tool for helpers for increasing coping with civilians in war and refugees.

Everly and Lating (2019), refer to six primary factors that may protect against and aid in recovery from extreme or traumatic stress and in building resilience: (1) actively facing fears and trying to solve problems; (2) regular physical exercise; (3) optimism; (4) following a moral compass; (5) promoting social support, nurturing friendships, and seeking role models; and (6) being open-minded and flexible in the way one thinks about problems, and avoiding rigid and dogmatic thinking. Using some of these components as helpers, we can promote people’s resilience by helping trauma-affected individuals to see their own strengths, to see whatever choices there may be in the situation, by supporting their trust in themselves to be able to influence their environment, by supporting their belief that they can tolerate and bear their own thoughts and emotions, by teaching them to talk supportively to themselves, and by encouraging them to seek companionship and support through relationships (Switchboard, 2019). As a helper, you can strengthen traumatized people’s sometimes-frail belief in humanity by being kind and to be trusted.

At the present, when the western world is more aware of mental health issues, we need to build proactive programs that can offer models for strengthening resilience. In the current model, we seek to give low-threshold support to people who have suffered war-related traumatic events, so that more people may be helped. This avoids the likelihood of refugees becoming chronically ill or unable to function. However, we believe that these helpful actions should be integrated into existing models in order to strengthen resilience and mental health of the trauma-affected individuals: Provide basic safety, with access to shelter, food, and water; to be met with respect, acceptance, solidarity and human kindness; possibility to be able to stay active and use one’s resources, not be left to long-term passivity in reception center (the limbo!); to be given some basic knowledge of normal reactions to trauma, and what one can do to alleviate stress and help oneself and others.

Resilience has to be seen as a two-way process in order to provide better support. In addition to providing a support mechanism for refugees, we also need to prepare our communities in a more sustainable way. Host communities need awareness and training for the communities, to allocate resources to those with an acute need for physical or mental health treatment or other psychosocial measures. For the communities to have a long-term perspective and preparedness to meet the mental health needs of the refugees would be vital. Such perspective and preparedness can help them in assessing what needs should have precedence. Some needs may arise during ongoing war danger, some needs may arise shortly after individual safety is restored, and some support needs might be more relevant during the process of limbo and waiting. Lastly, for some people, mental health problems and relevant support needs may become more relevant in the aftermath of trauma. Such needs may only arise or be recognized months or even years later.

#### Take a strength-based approach

When working with refugees, focusing on their strengths rather than their deficits can be helpful to increase resilience (Switchboard, 2019). There are 10 principles for implementing a strength-based approach. The first includes empowering, by focusing on their ability to identify strengths and solve problems. Second, be culturally humble. People’s own culture can be a strength in different ways. Third, build supportive relationships between staff and others in the community. In some cases community-based therapy can create a long-term support and empowerment (Gruner et al., 2020). Fourth, identify ways to increase and strengthen the level of support around the person. Fifth, expand community engagement and outreach efforts when recognizing their support network. Sixth, acknowledge the different social-political contexts and histories, and how this contributes to both strengths and challenges when resettling. Seventh, strengthen relationships outside the initial refugee resettlements. Eighth, partner up with other refugee families to learn from each other and together. Ninth, identify goals and create an action plan to meet their goals. Tenth, adapt services to focus on the family’s or individual’s strengths.

#### Collecting data to provide the best resources, help and therapies

When working with refugees, it can be helpful to collect data on the occurring mental health problems to get a better overview of diagnoses and develop proper interventions (Ventevogel et al., 2019). The Integrated Refugee Health Information System (iRHIS) is a tool to collect data on mental health, neurological health, and substance abuse developed by the United Nations High Commissioner for Refugees (UNHCR). The iRHS contains nine definitions of mental, neurological, and substance abuse (MNS): epilepsy/seizure, alcohol/substance use disorder; intellectual disability/developmental disorder; psychotic disorder (including mania); delirium/dementia; depression or other emotional disorder; other emotional complaints; medically unexplained somatic complaint; and self-harm/suicide. One can also add other disorders if appropriate. The users of the iRHIS, including healthcare staff in refugee health facilities, can use the MNS categories to make a diagnosis of a person seen during a consultation (Ventevogel et al., 2019).

### As a helper make sure you take care of yourself

It is important as a therapist to understand the individual’s feelings. However, it is important to not take on the emotions as your own or become too involved in the client’s concerns. This can make you feel overwhelmed and stressed by your work (World Health Organization, 2016; Bhui, 2022). Another way to help yourself is to speak with colleagues and your supervisor regularly. Schedule proper breaks between clients to do something that helps you (going for a walk, breathing, chatting with colleagues, etc.) and ask for help (e.g., talk to your supervisor) if you are experiencing distress or you find that your work is bothering you (Bhui, 2022). Supervisors or managers should consistently check in on their staff and provide a community of support, inclusiveness, and transparency (World Health Organization, 2015). If you work alone or in a smaller organization, it can be helpful to stay in touch with other organizations working with the same issues to share your experiences (World Health Organization, 2015).

It is also vital that as helpers we are aware of the politics of being helpers in humanitarian work. Elizabeth Cullen Dunn (2018) has examined how individuals and communities who have been displaced by war experience the humanitarian aid system. Dunn argues that the aid system is often focused on providing basic necessities, such as food and shelter, but that it is less effective in addressing the emotional, social, and cultural needs of people who have been displaced (Dunn, 2018). Therefore, helpers are advised to be more cognizant of the emotional, social, and cultural needs of people who are living in the limbo of the Ukraine war.

The war circumstances make helpers’ work more complex because foreign aid is not apolitical. War can propagate conflicting perceptions, relationships, and political interests between the donors, and the local authorities (Dunn, 2012; Dunn, 2014). Therefore, help and foreign aid is highly political and thus closely related to the humanitarian–development–security nexus, where separation between the apolitical and the political can no longer hold. The proliferation of various tools to address crises also contributes to the complexity of emergencies. Bureaucratization of help and aid work is often focused on predetermined programming solutions, rather than solutions stemming from the identified local problems (Dunn, 2012). Because of these complexities, mental health practitioners must continue to separate humanitarian work from political goals and integrate lived experiences of aid recipients to provide the support that would count as effective humanitarian work.

## Conclusion

The Ukraine war has created one of the largest exoduses of people from their homeland creating an enormous refugee crisis. While many European countries have accepted many refugees, the psychological limbo of being in and escaping the war continues. We conclude that the experience and effects of this limbo should be treated on multiple levels (physical, psychological, social, and institutional). Exposure to war trauma during childhood has long-term consequences that can persist into adulthood. The exposure to war trauma can interrupt life-long physical, mental, and emotional deficiencies. Research shows that trauma survivors can suffer from depression, anxiety, abandonment issues, unstable relationships, and other mental illnesses. Therefore, it is important for clinicians to understand better characterization of patient profiles and methodologies for successful treatment modalities to help alleviate the symptoms of trauma survivors. Clinicians must work collaboratively with patients regarding treatment preferences to ensure that the outcomes will be more effective and successful. This will help the helpers in providing a more nuanced framework for supporting refugees. There is a need to utilize experiential learning from the ongoing work with refugees of the Ukraine war to provide a better support infrastructure for being better prepared for working with war refugees in general.

In the end, we would like to acknowledge that there are two caveats to this paper. First, the review work presented in this paper is based on preliminary and initial work published on the effects of the Ukraine war. This literature is still in its infancy; therefore, we have capitalized on reviewing literature from other war contexts. We have applied the existing literature to develop a framework of living in limbo to highlight the multilevel effects of war on civilians who are internally displaced Ukrainians and Ukrainian refugees. The second caveat of this work is that the practical help strategies we have provided in this paper were accumulated for months specifically to help Ukrainian refugees in Norway and Canada. Indeed, many of these helping strategies are adapted from other war contexts and empirical research, which may be a limitation of this work. However, these strategies were recommended by our panel of psychologists and trauma therapists who have been approved for working with Ukrainian refugees. In our feedback sessions with more than one hundred Ukrainians, we have found positive feedback and endorsement of these strategies.

## Author contributions

GA was the lead author who conceptualized this project, prepared the manuscript and wrote the first two drafts of the paper. She received feedback from a clinical psychologist on the manuscript and integrated their feedback. She revised the manuscript and finalized the revision. MA was the second author who helped with the first and the second draft of the manuscript, and participated in the revision and finalization of the revision. HH worked on the first and second draft of the manuscript and approved the revision of the manuscript. All authors approved the revision and proofs of the manuscript.

## Funding

GA received financial support from the Department of Psychology (PSI), University of Oslo (UiO) for writing this paper. The funding was provided by Tildeling av kvalifiseringsstipend 2022, and Mobility grant PSI 2022, sub-project number: 102603241.

## Conflict of interest

The authors declare that the research was conducted in the absence of any commercial or financial relationships that could be construed as a potential conflict of interest.

## Publisher’s note

All claims expressed in this article are solely those of the authors and do not necessarily represent those of their affiliated organizations, or those of the publisher, the editors and the reviewers. Any product that may be evaluated in this article, or claim that may be made by its manufacturer, is not guaranteed or endorsed by the publisher.
